# Supplementary material for: The maternal vGluT2 and embryonic mGluR3 signaling relay system controls offspring wing dimorphism in pea aphid
Source: iScience. 2025 May 6;28(6):112591. doi: 10.1016/j.isci.2025.112591 (PMC12145829; doi:10.1016/j.isci.2025.112591)
Supplement: Document S1. Figures S1–S6 and Tables S1 and S2 [file mmc1.pdf]

iScience, Volume *28*

Supplemental information

The maternal vGluT2 and embryonic mGluR3 signaling relay system controls offspring wing dimorphism in pea aphid

Yiyang Yuan, Yanyan Wang, Wanwan Ye, Liqiang Xie, Erliang Yuan, Huijuan Guo, Shifan Wang, Fang Dong, Keyan Zhu-Salzman, Feng Ge, and Yucheng Sun

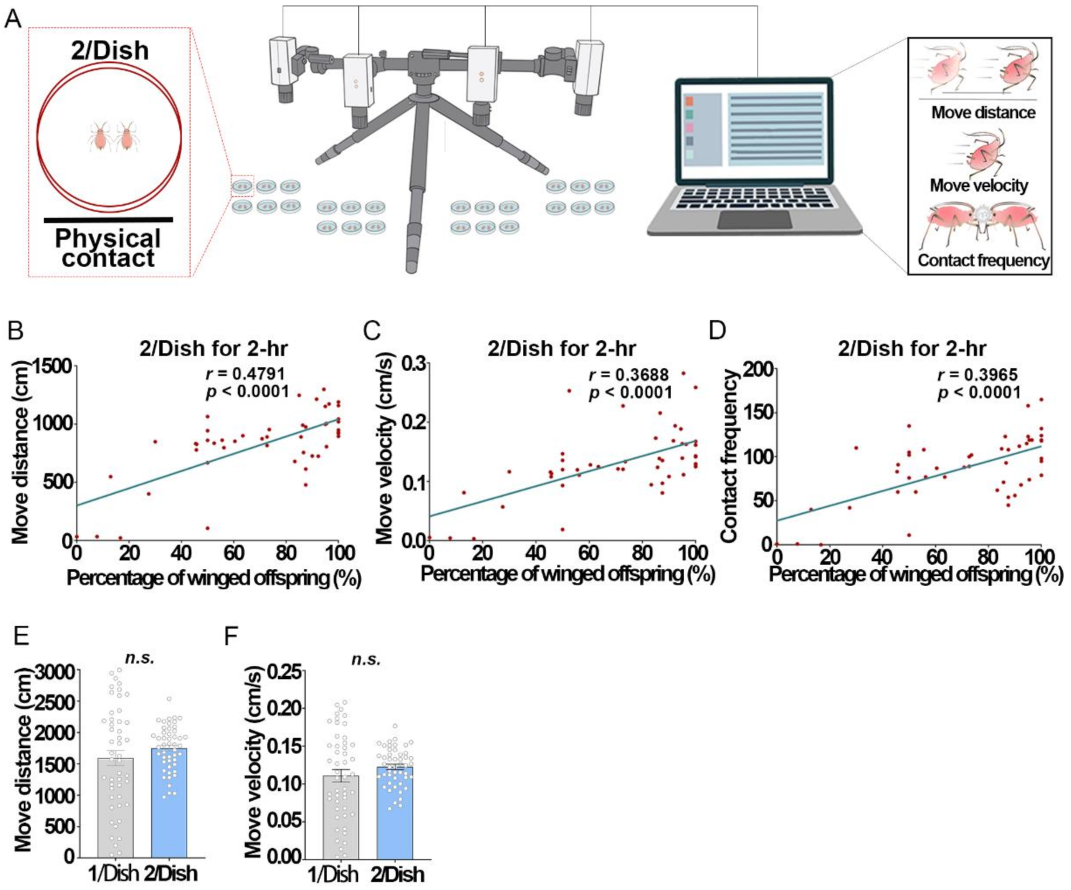

Figure S1 Correlation between the proportion of winged offspring and maternal locomotor activity. (A) Schematic diagram of locomotor activity monitoring. (B) Linear regression of the proportion of winged offspring vs the move distance, or (C) the move velocity, or (D) the contact frequency in maternal aphids. (E) The movement distance and (F) velocity of maternal aphids subjected to the two-adult contacting for 4-hr were similar to those of solitary maternal aphids. Student *t* test was used to compare means, n = 48, *n.s.* not significant.

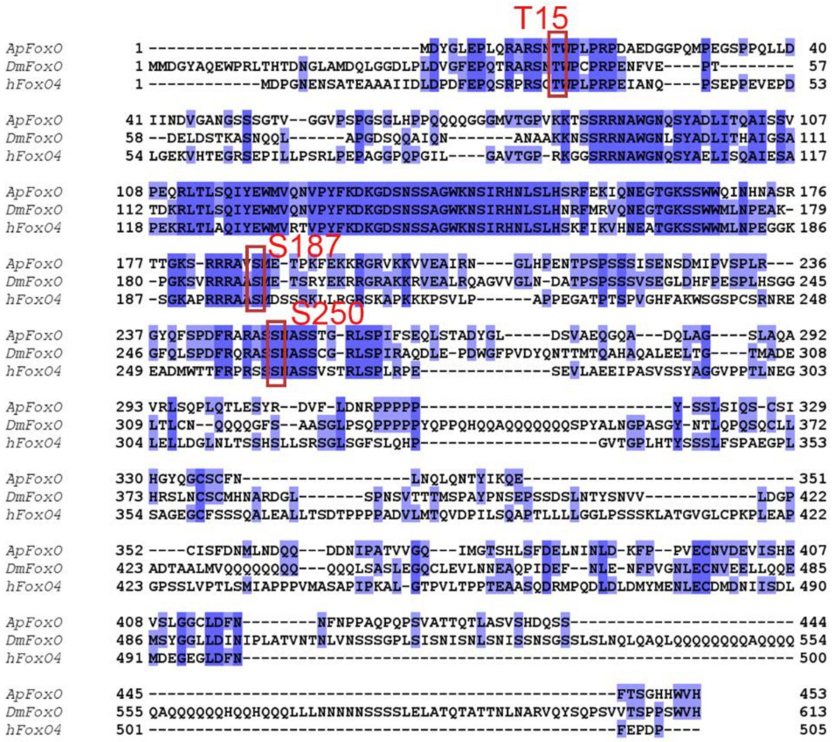

Figure S2 Multiple amide acid sequence alignment indicating pea aphid FoxO potentially shares three conserved phosphorylation sites with *Drosophila* FoxO and Human FoxO4. Three conserved phosphorylation sites, including T15, S187 and S250, were highlighted. *Ap*, *Acyrthosiphon pisum*; *Dm*, *Drosophila melanogaster*; *H*, *Homo sapiens*.

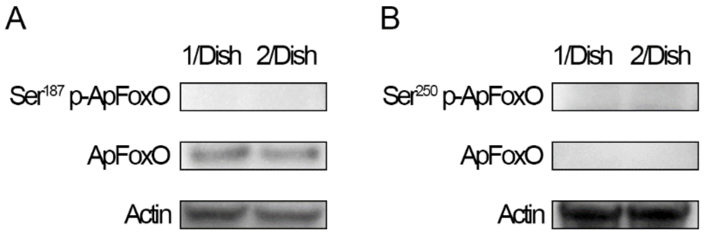

Figure S3 Evaluation of phosphorylated ApFoxO antibodies used in western blot targeting (A) phosphorylated S187-ApFoxO, and (B) phosphorylated S250-ApFoxO.

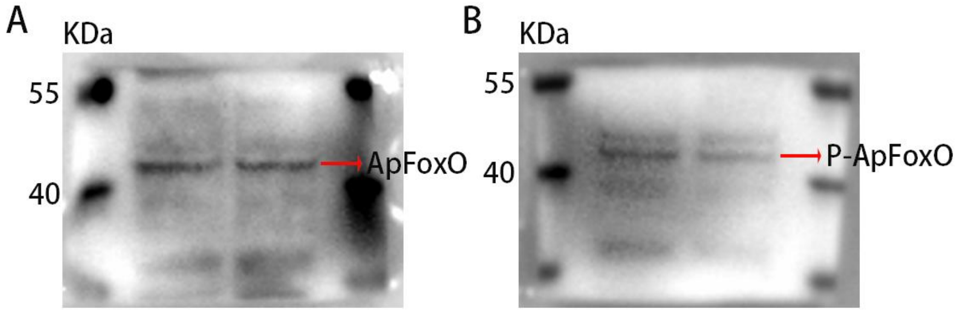

Figure S4 Representative images of original unprocessed Western blots used for detecting (A) ApFoxO and (B) phosphorylated T15-ApFoxO.

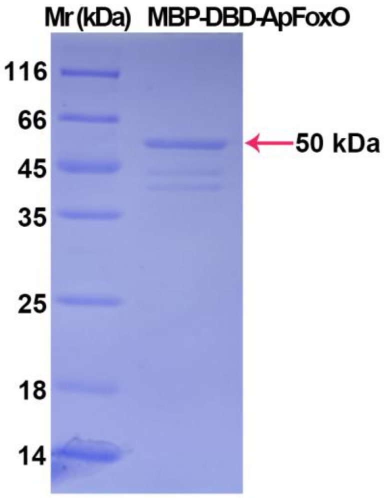

Figure S5 SDS-PAGE of purified MBP--DBD$_{ApFoxO}$ recombinant protein.

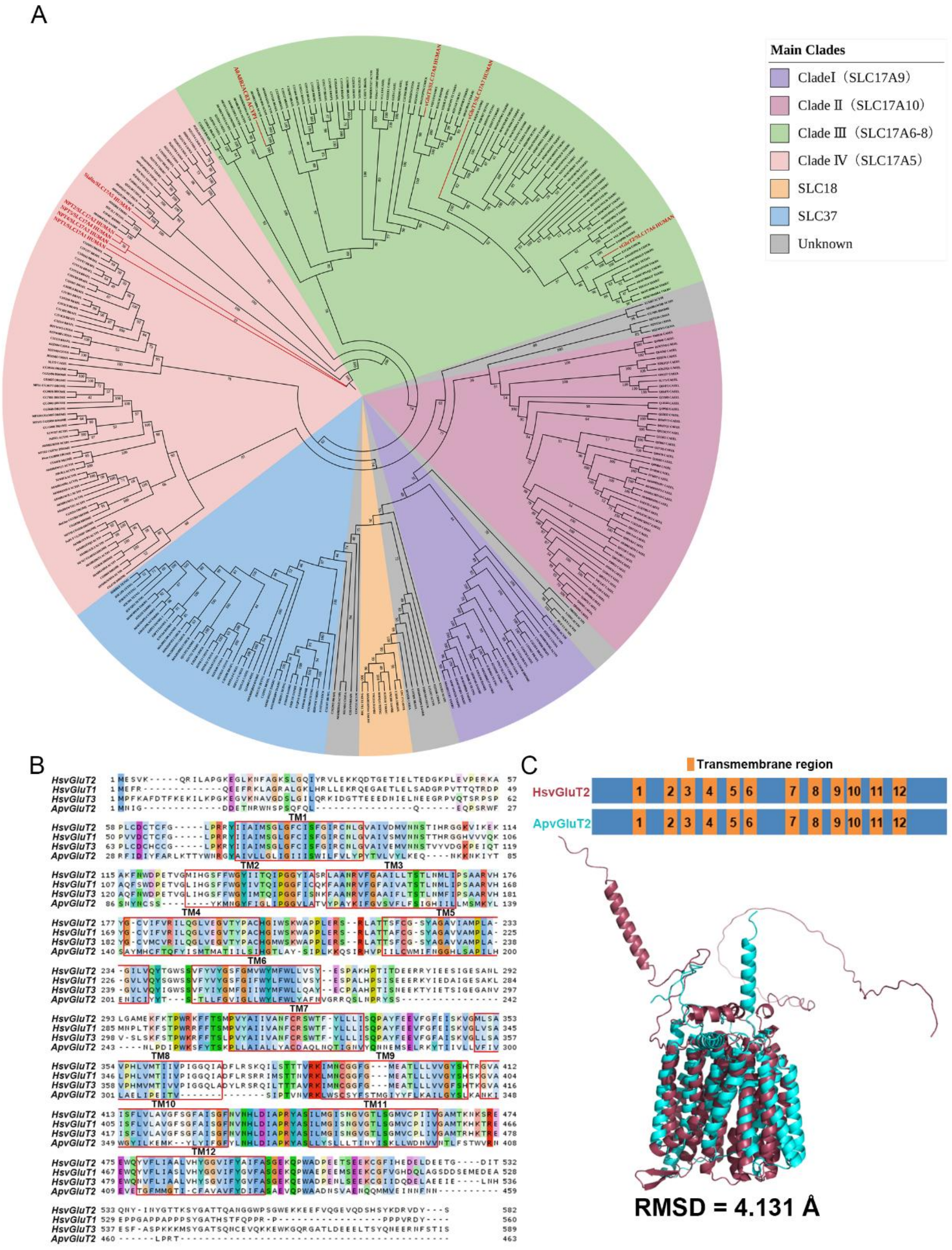

Figure S6 ApvGluT2 is a structurally conserved protein with 12 transmembrane domains. (A) The phylogenetic analysis of the vGluT2 family proteins indicated that ApvGluT2 belonged to the Clade III SLC17A9 subfamily. Bootstrap values expressed as percentage of 1000 replications were shown at branch points. Pea aphid vGluT2 (UniProt entry: A0A8R2AC83) and human homologs were highlighted by red

characters. (B) Multiple sequence alignment and (C) pairwise structural alignment indicated that ApvGluT2 containing 12 transmembrane domains were similar to human homologs. Red rectangles marked 12 transmembrane domains. The structure of ApvGluT2 was predicted by AlphaFold2. The pairwise structural alignment was performed by PyMOL software. TM, transmembrane domain. ACYPI, *Acyrthosiphon pisum*; BRAFL, *Branchiostoma floridae*; CAEEL, *Caenorhabditis elegans*; CHICK, *Gallus gallus*; CIOSA, *Ciona savigyni*; DANRE, *Danio rerio*; DROME, *Drosophila melanogaster*; HUMAN, *Homo sapiens*; TAKRU, *Takifugu rubripes*; TETNG, *Tetraodon nigroviridis*. RMSD, root mean square deviation.

Table S1 Primers for RT-qPCR

Gene name	Gene ID	Forward	Reverse
RPL27	ACYPI000085	5'–TCGTTACCCTCGGAAAGTC–3'	5'–GTTGGCATAAGGTGGTTGT–3'
vGluT2	LOC100168565	5'–AGGTTTAAGGATCAGCCCGC–3'	5'–GCCCCAAGATCCCTGGAAAT–3'
mGluR3	LOC107882629	5'–CCTCATATTGGACGACTGCGA–3'	5'–ACGCCTTTCGAAGGTTCTGT–3'
GluCl-α	LOC100162577	5'–CCATGAACGTTTGGGATGGC–3'	5'–ATTGGTAACGGCCAGTCTGA–3'
Octβ1R	LOC100166522	5'–AGTACGCTGTCGTGTCTTCC–3'	5'–GTGCTTGTTCAGTAACGCGG–3'
GLRA1	LOC100169196	5'–GGGTCCAACAAAGTTCACGC–3'	5'–GGTCCACTGTTCGTCGTCAT–3'
HTR2A	LOC100575043	5'–GGTCAGACGAGACGGACATC–3'	5'–GAATCGCCGTGACCCTTTTG–3'
FoxO	LOC100168097	5'–ACAGTTCGGCTGGATGGAAG–3'	5'–GGCTCGCGTTGTGGTTTATC–3'
Dpp	LOC100163532	5'–GTCGGTTGGGACGATTGGAT–3'	5'–TGGTGGAGTTGAGGTGTTCG–3'
Hh	LOC100165590	5'–GCGGGAGTGAAGAAGCAGAT–3'	5'–CACGTTACGGTCCAGGAACA–3'
Vg	LOC100570322	5'–ACCAACAAGCGTCCACATCT–3'	5'–CTGCTGTGGTGTGAGTCTGT–3'
Mats	LOC100160461	5'–CCACTCTTGGTTCGGGCAAT–3'	5'–CCTCGGTGCAGAACTCTGTT–3'

Table S2 Primers for dsRNA synthesis

Primer name	Gene ID	Forward	Reverse
GFP	-	5'–CACAAGTTCAGCGTGTCCG–3'	5'–GTTCACCTTGATGCCGTTC–3'
vGluT2	LOC100168565	5'–AGTGCTCCTATCCTCCACGA–3'	5'–AATATCGCGGGTTCAGGCTC–3'
mGluR3	LOC107882629	5'–GTAGTCGAGGGCGATTTGCT–3'	5'–ACAGATGTGACACTTGACGC–3'
FoxO	LOC100168097	5'–TTTCTCAGCCGCTACAGACG–3'	5'–GTCATCCTGCTGGTCGTTCA–3'
